# Teff Type-I Sourdough to Produce Gluten-Free Muffin

**DOI:** 10.3390/microorganisms8081149

**Published:** 2020-07-29

**Authors:** Cinzia Dingeo, Graziana Difonzo, Vito Michele Paradiso, Carlo Giuseppe Rizzello, Erica Pontonio

**Affiliations:** 1 Department of Soil, Plant and Food Science, University of Bari Aldo Moro, 70126 Bari, Italy; cinzia.dingeo@uniba.it (C.D.); graziana.difonzo@uniba.it (G.D.); carlogiuseppe.rizzello@uniba.it (C.G.R.); 2 Department of Biological and Environmental Sciences and Technologies, University of Salento, 73100 Lecce, Italy; vito.paradiso@unisalento.it

**Keywords:** type-I sourdough, teff, gluten-free, nutritional profile, celiac disease

## Abstract

The increasing number of persons following a gluten-free (GF) diet and the need for healthy and natural products are forcing researchers and industries to provide gluten-free products with high nutritional value. Here, a biotechnological approach combining the use of teff flour and type-I sourdough has been proposed to produce GF muffins with nutritional benefits. Teff-sourdough was prepared and propagated following the traditional daily refreshment procedure until the biochemical stability was achieved. The sourdough, dominated by *Lactiplantibacillus plantarum*, *Limosilactobacillus fermentum* and *Saccharomyces cerevisiae* strains, was used to produce muffins at three different levels (up to 15%, wt/wt) of fortification, achieving several positive effects on the nutritional properties of the products. The use of teff flour led to high content of fiber (>3 g/100 g) and proteins (>6 g/100 g) in muffins achieving the nutritional requirements for the healthy claims “source of fiber” and “rich in protein”. Thanks to their metabolic traits, sourdough lactic acid bacteria caused the increase of the total free amino acids (TFAA, up to 1000 mg/kg, final concentration) and phytic acid decrease (50% lower than control), which positively affect the nutritional properties of the products. Besides, high in vitro protein digestibility (IVPD, 79%) and low starch hydrolysis rate (HI, 52%) characterized the fortified muffins. Muffins also presented high in vitro antioxidant (56%) and mold-inhibitory activities, potentially contributing to an extended shelf-life of the products.

## 1. Introduction

Lately, there has been an increasing interest surrounding celiac disease (CD) due to a rise in diagnoses. Recent estimates indicate about 1–2% prevalence of CD worldwide [1]. Currently, there is no treatment available for the disease, other than following a lifelong strictly gluten-free diet [2]. With the advancement in scientific knowledge and processing technology, there has been tremendous growth in the number of gluten-free (GF) products and especially bakery products available to consumers in specialty stores and supermarkets [2]. Nonetheless, the concerns about the nutritional profile of the GF products persists due to a range of deficiencies, including fiber, protein, calcium, folate, iron and vitamins B12 and D [3,4,5]. Another important nutritional issue of GF foods is related to the relatively high glycemic index (GI), which is mainly affected by available carbohydrates but also depends on multiple ingredients, including fibers and proteins and fat [6].

Nevertheless, the development of GF products is still a technological challenge, the use of naturally GF ancient and minor cereal (e.g., teff, emmer, spelt) might be a valuable option due to their high nutritional value and functional properties. Indeed, these have been shown to be well suited to making highly nutritious, modern and innovative baked goods meeting functional and sensory standards in terms of nutritional added value, palatability, convenience (extended shelf life) and easy handling during processing.

Among all, teff (*Eragrostis tef* Zucc.) is gaining popularity around the world mostly due to its attractive nutritional properties [7]. Teff is a GF grain and has great potential to be formulated into a range of food/beverage products to aid people with celiac disease [7]. Due to the very small grain size, teff milling necessarily provides a whole grain flour. Therefore, the flour is rich in fiber due to the incorporate of the bran components. It is also a source of bioactive compounds such as polyphenols [8]. As a result of the unique chemical composition and the whole grain form, a range of health benefits have been associated with teff that is, in vitro anti-oxidative activities and prevention of incidence of anemia and diabetes [7,9]. Despite the high nutritional value, the high content of dietary fiber, the presence of the anti-nutritional factors (e.g., phytic acid) and the absence of gluten might represent a limit to produce bakery products with acceptable technological, nutritional and sensory profiles [10].

The sourdough technology has largely been proposed as tool to overcome such drawbacks related to the use of wheat-alternatives flours in bakery production thanks to the direct and indirect activity of the autochthonous microbiota composed of lactic acid bacteria (LAB) and yeast [11].

The present study aimed at optimizing a biotechnological protocol to produce a gluten-free muffin with high nutritional value and optimal technological and sensory properties. Thus, teff type-I sourdough has been produced and propagated till the biochemical stability was achieved. Biochemical and microbiological properties of sourdough were investigated. The sourdough was used to fortify teff muffins. The biochemical, nutritional, structural and sensory characteristics of the fortified muffins were evaluated and compared to those of a muffin produced without sourdough.

## 2. Materials and Methods

### 2.1. Teff Sourdough

#### 2.1.1. Propagation

Sourdough was prepared and propagated according to the traditional protocol commonly used for wheat sourdough [12]. The dough used for starting sourdough preparation was made with teff flour (Maiglù s.r.l., Altamura, Italy) (166.6 g) and tap water (133.3 mL) (dough yield, DY [dough weight∗100/flour weight], 180), mixed (60 g for 5 min) with a continuous high-speed mixer (Chopin & Co., Boulogne sur Seine, France). Sourdough propagation was carried out according to the back-slopping (refreshment) procedure and without using starter cultures or baker’s yeast. In detail, the sourdough from the day before was used as the starter (25% [wt/wt] of inoculum) to ferment a new mixture of flour (125 g) and tap water (100 mL), having a DY of 180. First incubation lasted 24 h at 30 °C, while following incubations lasted 8 h at 30 °C. Three batches of sourdough were prepared, and each batch was daily propagated for 12 days. Samples (three aliquots/each batch) were taken after 0 (dough), 1, 3, 5, 7, 9, and 12 (sourdough) days of propagation. Sourdough aliquots were cooled down to 4 °C and analyzed within 2 h after collection.

#### 2.1.2. Chemical and Microbiological Characterization

The values of pH were determined by a pH-meter (Model 507, Crison, Milan, Italy) with a food penetration probe. Total titratable acidity (TTA) was determined on 10 g of sourdough homogenized with 90 mL of distilled water and expressed as the amount (mL) of 0.1 M NaOH to reach pH of 8.3.

Water/salt-soluble extracts (WSE) from dough and sourdoughs were prepared and used to analyze organic acids and total free amino acids (TFAA). Organic acids were determined by High Performance Liquid Chromatography (HPLC), using an ÄKTA Purifier system (GE Healthcare, Buckinghmshire, UK) equipped with an Aminex HPX-87H column (ion exclusion, Biorad, Richmond, CA, USA) and a UV detector operating at 210 nm. TFAA were analyzed by a Biochrom 30 series Amino Acid Analyzer (Biochrom Ltd., Cambridge, UK) with a Na-cation-exchange column (20 by 0.46 cm internal diameter) as previously reported by Pontonio et al. [13]. The quotient of fermentation (QF) was determined as the molar ratio between lactic and acetic acids.

For microbiological analyses, 10 g of dough and sourdoughs were suspended in 90 mL of sterile sodium chloride (0.9%, wt/vol) solution and homogenized in a Stomacher lab blender for 2 min at room temperature. Presumptive LAB were determined on De Man, Rogosa and Sharpe (MRS, Oxoid, Basingstoke, Hampshire, UK) supplemented with cycloheximide (0.1 g/L), at 30 °C for 48 h under anaerobiosis. Molds were enumerated on Potato Dextrose Agar (PDA, Oxoid) at 25 °C for 48 h. The cell density of yeasts was estimated on Sabouraud Dextrose Agar (SDA, Oxoid), supplemented with chloramphenicol (0.1 g/L) at 25 °C for 48 h. Total *Enterobacteriaceae* were determined on Violet Red Bile Glucose Agar (VRBGA, Oxoid) at 37 °C for 24 h and total mesophilic bacteria were determined on Plate Count Agar (PCA, Oxoid) at 30 °C for 48 h.

#### 2.1.3. Isolation, Genotypic Characterization, and Identification of Lactic Acid Bacteria and Yeasts

LAB and yeasts were isolated from dough (t0) and mature sourdough (t12). At least 20 colonies of presumptive LAB were randomly selected from the plates containing the two highest sample dilutions. Gram-positive, catalase-negative, non-motile rod and coccus isolates were cultivated in MRS (Oxoid) broth at 30 °C for 24 h and re-streaked onto the same agar medium. All isolates considered for further analysis were able to acidify the culture medium. Similarly, at least 20 colonies of yeasts isolated from the mature sourdough were sub-cultured in SDA and re-streaked onto the same agar media.

Genomic DNA of LAB and yeasts was extracted using a DNeasy blood and tissue kit (Qiagen, SA, Courtaboeuf, France) and Wizard Genomic DNA Purification Kit (Promega) respectively, according to the manufacturer’s instructions.

Oligonucleotides, P4, P7 and M13 and M13m and Rp11, with arbitrarily chosen sequences, were respectively used for bio-typing of LAB and yeasts [14]. RAPD-PCR (Randomly Amplified Polymorphic DNA) profiles were acquired by the MCE-202 MultiNA microchip electrophoresis system (Shimadzu s.r.l., Milan, Italy), using the DNA-2500 reagent kit (100–2500 bp) and the 2-log DNA ladder (0.1–10.0 kb) (Promega Srl, Padova, Italy) according to the manufacturer’s instructions. RAPD-PCR was also applied to identify unique populations. The similarity of the electrophoretic profiles was assessed by determining the Dice coefficients of similarity and using the unweighted-pair group method using average linkages (UPGMA) algorithm.

To identify presumptive LAB, two primer pairs (Invitrogen Life Technologies, Milan, Italy), LacbF/LacbR and LpCoF/LpCoR, were used for amplifying the 16S rDNA [15]. Primers designed for the recA gene were also used to distinguish *Lactiplantibacillus plantarum* subsp. *plantarum*, *Lactiplantibacillus pentosus* and *Lactiplantibacillus paraplantarum* species [16]. To identify presumptive yeasts two primers NL-1 (5′-GCATATCAATAAGCGGAGGAAAAG-3′) and NL-4 (5′-GGTCCGTGTTTCAAGACGG-3′) were used for amplifying the divergent D1/D2 domain of the 26S rDNA [17].

PCR products were separated by electrophoresis on an agarose gel at 1.5% (wt/vol) (Gellyphor; EuroClone) and amplicons were purified by the Nucleospin gel and PCR clean-up kit (Macherey–Nagel, Düren, Germany) and subjected to Sanger sequencing [14]. rRNA sequence alignments were carried out using the multiple-sequence alignment method [18] and identification queries were fulfilled by a BLAST search [19] in GenBank (http://www.ncbi.nlm.nih.gov/GenBank/).

Strains showing homology of at least 97% were considered to belong to the same species [20]. Cultures of LAB and yeast were maintained as stocks in 15% (vol/vol) glycerol at −80 °C and routinely propagated at 30 °C for 24 h in MRS and SDA broth, respectively.

#### 2.1.4. Antioxidant Activity

The 2,2-diphenyl-1-picrylhydrazyl (DPPH) radical scavenging activity was determined on the methanolic extract (ME) and WSE of type-I sourdough (t12). For the ME, 3 g of each sample were mixed with 30 mL of methanol (80%, vol/vol). The mixture was purged with nitrogen stream for 30 min, under stirring condition and centrifuged at 4600× *g* for 20 min. The supernatants (MEs) were transferred into test tubes, purged with nitrogen stream and stored at ca. 4 °C before analysis. The radical DPPH˙ was used for determining the free radical scavenging activity [21]. The synthetic antioxidant butylated hydroxytoluene (BHT) was included in the analysis as the reference (75 ppm). Total phenols were determined on the ME as described by Slinkard and Singleton [22] and expressed as a gallic acid equivalent.

### 2.2. Muffin Preparation

Muffins were manufactured at the pilot plant of the Department of Soil, Plant and Food Science (University of Bari, Italy) according to a procedure resembling the two-stage protocol commonly used for typical Italian sourdough breadmaking. The protocol was adapted to muffins and included the production of the type I-sourdough (step I) and subsequent mixing at 5, 10, and 15% of fortification (wt/wt) (M_5%_, M_10%_ and M_15%_) of the final formulation (Table S1, step II). Solid ingredients were mixed first for one minute in a kneader (Kenwood, Hampshire, UK) and added to liquids. The final mixing, made at speed level n. 3, lasted 5 min. Fifty grams of dough were placed in each cup and baked in a preheated oven at 180 °C for 20 min. A batch of muffins without the type-I sourdough (M_CT_) was manufactured and used as the control. The muffins were marked and allowed to cool for 120 min on cooling racks at room temperature.

### 2.3. Muffin Characterization

#### 2.3.1. Chemical and Nutritional Characteristics

The values of pH and TTA, the concentration of organic acids, TFAA and total phenols and radical scavenging activity were determined as reported above.

Protein (N × 5.7), ash and moisture contents were determined according to the American Association of Cereal Chemists (AACC, 2000) approved methods 46–11.02, 44–19 and 08–01, respectively [23]. Total dietary fiber (TDF) was determined by the enzymatic-gravimetric procedure according to the method 991.43 (AOAC, 2005) [24]. Carbohydrates were calculated as the difference [100 − (moisture + proteins + lipids + ash + total dietary fiber)]. For the peptides analysis, WSE were treated with trifluoroacetic acid (0.05% wt/vol) and centrifuged (10,000× *g* for 10 min) to remove proteins. Then, samples were transferred into dialysis tubes (cut-off 500 Da, Fisher Scientific, Rodano, Italy) and dialyzed against water (1 L per 5 mL of sample) at 4 °C for 48 h to remove FAA. Retentates were freeze-dried and then resuspended in 50 mM Tris–HCl (pH 8.8). Then peptide concentration was determined by the o-phtaldialdehyde (OPA) method, as described by Church et al. [25]. All analyses were carried out in triplicate. 

#### 2.3.2. Fatty Acid (FA) Composition

The determination of the FA composition was carried out on lipid fraction extracted from muffins through the Soxhlet extraction according to the method 920.39 (AOAC, 2005) [24]. FA profiles were determined according to the official methods of European Communities 2568/91 [26]. The gas-chromatographic analysis of FA methyl esters was performed using C15:0 methyl ester as internal standard as described elsewhere [27]. In particular, the extracted fat was solubilized in 1 mL of hexane. Then, 2 μL of the hexane fraction was injected. The gas-chromatograph system was composed by an Agilent Technologies 7890 GC System (Agilent Technologies Inc., Santa Clara, CA, USA), equipped with a FID detector and a SPTM 2340 fused silica capillary column (Supelco, Bellefonte, PA, USA), 60 m length × 0.25 mm i.d. and 0.20 μm film thickness. The temperature of the split injector was 210 °C, with a splitting ratio of 1:100; the detector temperature was 220 °C. The oven temperature was gradually increased from 160 to 240 °C. Helium was used as carrier gas at a flow of 1 mL/min.

#### 2.3.3. Phytic Acid, Protein, and Starch Digestibility

Phytic acid concentration was measured using K-PHYT 05/07 kit assay (Megazyme Intl., Wicklow, Ireland), following the manufacturer’s instructions.

The in vitro protein digestibility (IVPD) of muffins was determined according to Akeson and Stahmann [28] with some modifications [29]. IVPD was expressed as the percentage of the total protein solubilized after a sequential enzymatic treatment mimicking the in vivo digestion enzymatic hydrolysis. The protein concentration was determined following the Bradford method [30].

The starch hydrolysis (HI) degree was determined according to the method (mimicked the in vivo digestion) proposed by De Angelis et al. [31]. The glucose released after the enzymatic process was measured with D-Fructose/D-Glucose Assay Kit (Megazyme, Wicklow, Ireland). HI was expressed as the percentage of potentially available starch hydrolyzed after 180 min. Wheat flour bread (WB) leavened with baker’s yeast was used as the control to estimate the hydrolysis index (HI = 100). The predicted GI (pGI) was calculated using the equation—pGI = 0.549 × HI + 39.71 [32].

#### 2.3.4. Structure and Color Parameter

Instrumental Texture Profile Analysis (TPA) was performed on the sliced muffin (150 mm thick) according to Pasqualone et al. [33]. A Z1.0 TN texture analyzer (Zwick Roell, Ulm, Germany), equipped with a stainless-steel square probe (4 cm side) and a 50 N load, cell was used. Data were acquired by the TestXPertII version 3.41 software (Zwick Roell, Ulm, Germany). The TPA conditions in the cyclic compression test were—(i) 1 mm/s probe compression rate; (ii) 40% sample deformation in both the compressions; and (iii) 5 s pause before second compression. The analyses were carried out in triplicate.

The image analysis was performed according to Scheuer et al. [34] with some modifications. The samples were cut into two halves and the picture of crumb was acquired using an Image Scanner (Amersham Pharmacia Biotech, Uppsala, Sweden) in full-scale mode, at 300 dots/in and elaborated through the ImageJ software (National Institutes of Health, Bethesda, Rockville, MD, USA). The procedure divides the image into object and background by obtaining an initial threshold value and computing averages for the pixels at or below the threshold and for those above as already reported elsewhere [34]. The images were converted into 8-bit greyscale and cropped for a section of 25 × 25 mm from the center of the product, then they were subjected to thresholding function to obtain the best cell resolution. The parameters were chosen to detect the cells with an area >0.05 mm^2^.

Colorimetric readings on crust and crumb were taken using a Minolta Chroma meter CR-300 (Osaka, Japan) with a CR 300 measurement head and CIE Standard Illuminant D65. Lightness (L*), redness (a*, ±red-green) and yellowness (b*, ±yellow-blue) were determined as color coordinates and their values are the differences between sample and reference a white ceramic plate having L = 67.04, a = 2.44 and b = 18.28.

#### 2.3.5. Sensory Flash Profiling

A Flash Profile (FP) sensory evaluation was carried out according to Liu et al. [35]. Twelve assessors were selected, without a previous specific training. As first step, the assessors were given an explanation about the procedure. Then, all assessors were asked to individually generate the sensory attributes that better described the differences among the samples and were instructed to avoid the use of hedonic terms. The list of the attributes chosen by the panel are reported in Table S2. Afterwards, it was requested to rate the samples using an unstructured linear scale. According to the IFST Guidelines for Ethical and Professional Practices for the Sensory Analysis of Foods, assessors gave informed consent to tests and could withdraw from the panel at any time, without penalty or having to give a reason.

#### 2.3.6. Volatile Compounds

Volatile compounds of the muffins were determined by headspace solid phase micro-extraction (HS-SPME) coupled with gas-chromatography/mass spectrometry (GC–MS) [36,37]. The samples were weighed (500 ± 0.05 mg) in a 12-mL vial and added of 100 μL of 1-propanol solution as internal standard plus 4 mL of saturated aqueous solution of NaCl. Vials were sealed by butyl rubber septa and aluminum crimp caps. Before volatile extraction, the mixture was homogenized for 2 min by using a laboratory vortex shaker. The extraction of volatile compounds was carried out by exposing a 75 μm Carboxen/polydimethylsiloxane (CAR/PDMS) SPME fiber (Supelco, Bellefonte, PA, USA) in the headspace of the sample at 40 °C for 50 min. The fiber was then desorbed for 6 min in the injection port of the gas-chromatograph, operating in split-less mode, at 230 °C for 3.5 min. An Agilent 6850 gas-chromatograph equipped with an Agilent 5975 mass-spectrometer (Agilent Technologies Inc., Santa Clara, CA, USA) was used. The volatile compounds were separated on a HP-Innowax (Agilent Technologies Inc., Santa Clara, CA, USA) polar capillary column (60 m length × 0.25 mm i.d. × 0.25 μm film thickness), under the following conditions—injector temperature, 250 °C; flow of 1.5 mL/min, pressure of the carrier (helium) 30 kPa. The oven temperature was held for 5 min at 35 °C then increased by 5 °C/min to 50 °C and held in isothermal conditions for 5 min, then raised to 210 °C at 5.5 °C/min and finally held constant at 210 °C for 5 min. The mass detector was set at the following conditions—interface temperature 230 °C; source temperature 230 °C; ionization energy 70 eV; scan range 33–260 amu. The volatile compounds were quantified by standardizing the peak areas of compounds of interest with the peak area of the internal standard (1-propanol). Peak identification was performed by computer matching with the reference mass spectra of National Institute of Standards and Technology (NIST) and Wiley libraries and by comparison of retention indices. The analyses were carried out in triplicate.

### 2.4. Bio-Preservation Effect of Teff Sourdough

The potential effect of the teff sourdough on the microbiological shelf-life of muffins was investigated through monitoring the fungal growth on sample surface. *Penicillium roqueforti* DPPMAF1 (belonging to the Culture of the Department of Soil, Plant and Food Science of the University of Bari) was used as an indicator mold. In details, each muffin was sliced after baking and cooling at room temperature (2 h). Slices (10 cm height and 1.5 cm width) were nebulized with a suspension of 10^2^ conidia/mL of *P. roqueforti* DPPMAF1 (I), obtained according the protocol previously described by Coda et al. [38]. Slices were packed in polyethylene bags to maintain constant moisture and incubated at room temperature for 21 days. Not inoculated (NI) slices were used as control. The analyses were carried out in triplicate.

### 2.5. Statistical Analysis

Three batches of sourdough were prepared and propagated. Each sourdough was used at three different levels of fortification to produce gluten-free muffins. Hence, three batches of fortified (M_5%_, M_10%_, M_15%_) and one of control (M_CT_) muffins were made. All the analyses were carried out in triplicate for each batch of sourdough and muffins (total of nine replicates). Data were subjected to one-way ANOVA; pair-comparison of treatment means was achieved by Tukey’s procedure at *p* < 0.05, using the statistical software, Statistica 12.5 (TIBCO Software Inc., Palo Alto, CA, USA) for Windows. Principal Component Analyses was used to elaborate data of the flash sensory profiling analysis.

## 3. Results

### 3.1. Teff Type-I Sourdough: Microbiological and Biochemical Characterization

**Figure 1:**
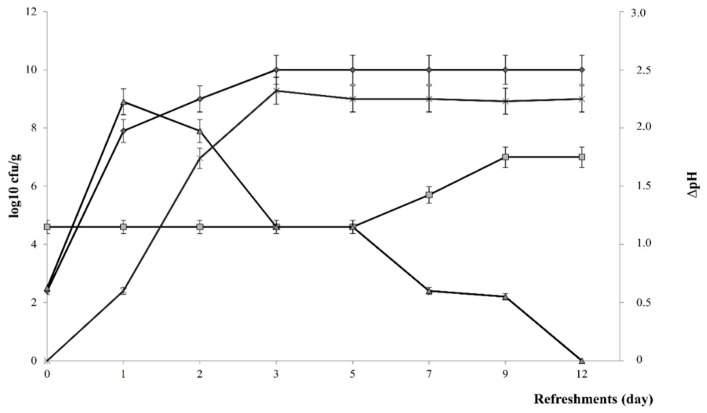
Cell density (log10 colony forming units (cfu)/g) of presumptive lactic acid bacteria (circle), yeasts (square) *Enterobacteriaceae* (triangle) and kinetic of acidification (star) of the teff type-I sourdough. Sourdough was daily propagated for twelve days and 0, 1, 3, 5, 7, 9, and 12 identified the dough (after mixing and before fermentation) and sourdough after one, two, five and ten days of propagation. Data are the means of three independent batches analysis ± standard deviations (*n* = 3). Bars of standard deviations are also represented.

Teff dough (t0) harbored *ca*. 2.5 and 4.6 log10 ufc/g *Enterobacteriaceae*, yeasts and presumptive LAB, respectively (Figure 1). After 24 h of fermentation (t1) the number of both presumptive LAB and *Enterobacteriaceae* increased significantly reaching values of *ca*. 8 log10 cfu/g. From the first (t2) to the third (t3) refreshment the cell density of presumptive LAB was subjected to a further increase and then remained stable till the end of propagation (t12). On the contrary, the number of *Enterobacteriaceae* decreased through the propagation till disappearing (Figure 1). Dough contained a significantly higher (*ca*. 2.0 log units) initial number of yeasts than of presumptive LAB (Figure 1). Nevertheless, yeast numbers remained stable until t5, then increased progressively until t9 reaching a constant value of 6.8 ± 0.1 log10 ufc/g. The ratio between LAB and yeasts stabilized to *ca*. 100:1 after 9 days of propagation.

Presumptive LAB (45 isolates) and yeasts (20 isolates) obtained from dough (t0) and sourdoughs (t12) were genetically characterized and identified through RAPD-PCR analysis and sequencing of the 16S/26S genes, respectively. *Lactiplantibacillus plantarum* (formerly known as *Lactobacillus plantarum*, 6 biotypes), *Limosilactobacillus fermentum* (formerly known as *Lactobacillus fermentum*, 1) [39] and *Saccharomyces cerevisiae* were identified as the dominant species.

During sourdough propagation, the median values of ΔpH ranged from 0 (t0) to 2.25 ± 0.03 (t12). TTA value significantly increased from *ca*. 2.2 ± 0.4 (t0) to ca. 15 ± 0.2 during the first seven refreshments (t7), then TTA remained almost constant.

The content of lactic and acetic acids in the dough prior the fermentation (t0) was not detectable. After 24 h of fermentation (t1) lactic and acetic acids values were 7.27 ± 0.05 and 2.7 ± 0.1 mmol/kg, respectively. Although the content of lactic acid increased progressively till the t3 remaining stable until the end of the propagation (t12), the concentration of acetic acid needed nine refreshment to stabilize. Mature sourdough (t12) contained 71.9 ± 0.3 and 27.5 ± 0.3 mmol/kg of lactic and acetic acids, respectively. The QF stabilized to *ca*. 3 after 9 days of propagation.

Dough prior the fermentation was characterized by a concentration of TFFA of 1577 ± 20 mg/kg. After first fermentation (t1) an increase of *ca*. 106% was found with a value of 3250 mg/kg. This value remained almost stable till the t9. After that, a further increase (*ca*. 40%) was found; indeed, mature sourdough (t12) contained 4622 ± 48 mg/kg. Moreover, mature sourdough (t12) contained 3.89 ± 0.04 mmol/kg of phenols and was characterized by a radical scavenging activity of 56 ± 0.1%.

### 3.2. Teff Muffins

#### 3.2.1. Biochemical and Nutritional Characteristics

Table 1 summarizes the proximate composition on the muffins. Significant differences were found for moisture, carbohydrates and ash contents. The former was significantly higher in M_10%_ and M_15%_ as compared to M_CT_ and M_5%_. Carbohydrates followed opposite trend; indeed, the content seemed to be slightly higher in M_CT_ and M_5%_. Overall, higher content of nutritionally valuable unsaturated (PUFA) than monounsaturated (MUFA) fatty acids were found in all muffins. Oleic and linoleic acid accounted for 36 and 51% of the total fatty acids (data not shown). Moreover, ash content was significantly lower in M_5%_ as compared to other sourdough muffins and control.

Biochemical characteristics of the muffins are shown in Table 2. All fortified muffins had a value of pH significantly lower than the M_CT_, however the magnitude of difference was in accordance to the level of fortification. Indeed, M_15%_ was characterized by the lowest value. On the contrary, the value of TTA increased according to the level of fortification. Overall, higher values (up to *ca*. 3-times) of TTA were found in fortified muffins as compared to M_CT_. High acidity was mainly due to the content of organic acids. Indeed, the concentrations of lactic and acetic acids were in the range of 25.9 ± 0.4–48.4 ± 0.6 and 9.8 ± 0.3–20.8 ± 0.4 mmol/kg, respectively. The concentration of both organic acids increased according to the level of fortification. M_15%_ contained a concentration of lactic acid from 6 to 10% higher than M_10%_ and M_5%_, respectively (Table 2). Similarly, the content of acetic acid in M_15%_ was *ca*. 2-times higher than M_5%_ and M_10%_. The QF was not significantly different among samples.

**Figure 2:**
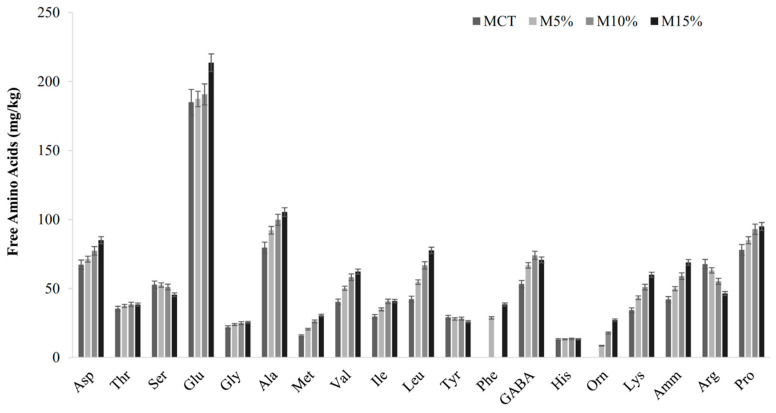
Concentration of free amino acids and amino acid derivatives (mg/kg) in teff muffins: M_5%_, muffin containing 5% (wt/wt) type-I sourdough; M_10%_, muffin containing 10% (wt/wt) type-I sourdough; M_15%_, muffin containing 15% (wt/wt) type-I sourdough; M_CT_, muffin made without type-I sourdough. Data are the means of three independent batches analysis ± standard deviations (*n* = 3). Bars of standard deviations are also reported.

Fortified muffins contained level of TFAA from *ca*. 2- to 3- times higher than M_CT_ (Table 2), being the highest in M_15%_. Except for threonine (Thr), serine (Ser), glycine (Gly), tyrosine (Tyr) and histidine (His), the concentration of the single amino acids and their derivatives were subjected to changes as results of the fortification (Figure 2). Overall, the content of amino acids increased according to the level of inoculum. Methionine (Met), valine (Val), leucine (Leu) and lysine (Lys) were from ca. 54 to 94% higher in M_15%_ than M_CT_ (Figure 2). Glutamine (Glu) was the most abundant (>150 mg/kg) amino acid in all samples. Contrarily, the concentration of arginine (Arg) was lower in fortified muffins as compared to M_CT_. Higher concentrations of peptides were found in fortified muffins as compared to the control. Indeed, concentrations of peptides were 50 (M_5%_ and M_10%_) and 75% (M_15%_) higher in fortified muffins as compared to M_CT_ (Table 2).

#### 3.2.2. Total Phenols and Antioxidant Activity

The concentration of total phenols in the ME was significantly higher (17–62%) in fortified muffins as compared to M_CT_. Similar trend was found for the scavenging activity. Indeed, 43–60% and 10–25% higher values (than control) were found in the ME and WSE, respectively. The increase was in accordance with the level of inoculum and the content of phenols and peptides, respectively (Table 2).

#### 3.2.3. Phytic Acid, IVPD and Starch Hydrolysis

Lower contents of phytic acid were found in fortified muffins as compared to the M_CT_. Values ca. 48% lower were found in M_5%_ and M_10%_, while M_15%_ contained 57% lower content of phytic acid compared to M_CT_ (Table 3). On the contrary, higher values of IVPD, from 40 (M_5%_) to 56% (M_15%_) were found in fortified muffins as compared to M_CT_. No significant differences were found among M_5%_ and M_10%_ (Table 3). Although no significant difference was found among M_CT_ and M_5%_ in terms of HI, slight but significant lower values were found in M_15%_ (*ca*. 20% lower). Similar trend was found for pGI (Table 3).

#### 3.2.4. Volatile Components

Table 4 shows the main classes of volatile compounds detected in the samples. Two of the most abundant compounds are nonanal and 1-hexanol, which showed significant higher concentrations in fortified muffins than M_CT_. A similar trend was found for the aldehydes and alcohols. In detail, the concentration of phenylethyl alcohol and phenylacetaldehyde was up to 4-times higher in fortified muffins as compared to M_CT_. Among the furan compounds furan, 2-penthyl- and ethyl-octanoate were more abundant in the samples with type-I sourdough. Moreover, the addition of type-I sourdough did not affect the concentration of ketones.

### 3.3. Structural Properties and Sensory Profile of the Teff Muffins

Structural properties of the muffins were slightly affected by the substitution, indeed only firmness and cohesiveness showed significant differences between samples. While M_10%_ and M_15%_ were characterized by a firmness lower than the control (M_CT%_), M_5%_ showed a value significantly higher than M_CT%_ and muffins with higher level of substitution. As compared to other samples, significantly higher value of cohesiveness was found for M_10%_ (Table 5). The higher was the inoculum of type-I sourdough the higher and lower were the mean area and the cell density, respectively. Moreover, according to the image analysis, small and medium pore did not show a specific trend whereas large pores, with cell area 8–16 and 16–30 mm^2^, were found only in the crumb of the samples with type-I sourdough and especially in the samples M_10%_ and M_15%_ (Figure S1).

**Figure 3:**
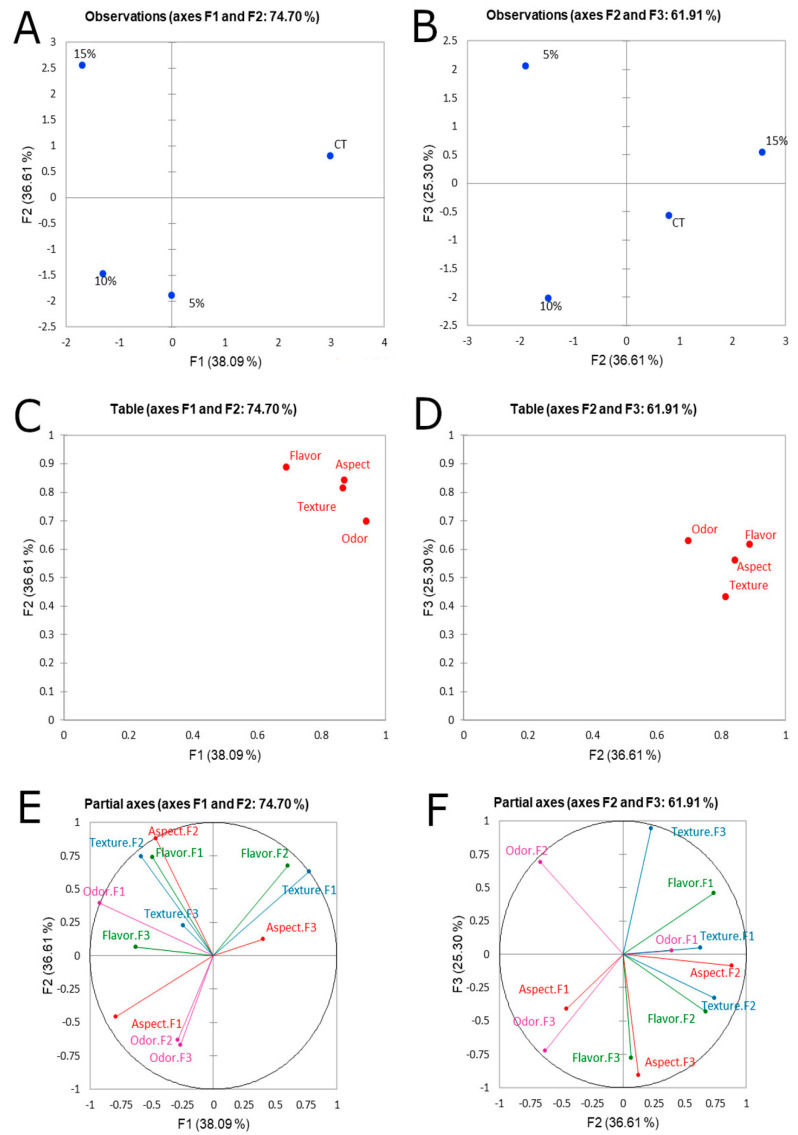
Multifactorial analysis (PCA) on the muffin sensory attributes grouped in odor, taste, texture and appearance. M_5%_, muffin containing 5% (wt/wt) type-I sourdough; M_10%_, muffin containing 10% (wt/wt) type-I sourdough; M_15%_, muffin containing 15% (wt/wt) type-I sourdough; M_CT_, muffin made without type-I sourdough. (**A**,**C**), score plots of PC1 vs. PC2; (**B**,**D**) score plots of PC2 vs. PC3; (**E**) loading plot of PC1 vs. PC2; (**F**), loading plot of PC2 vs. PC3.

An FP sensory analysis has been performed to define the sensory profile of the muffins (Figure 3A–F). The data collected were statistically analyzed through the multifactorial Principal Component Analysis (PCA). According to the sample’s distribution on the plane, the PC1 discriminates the M_CT_ from the fortified muffins, however, PC2 allows the separation between M_15%_ and the other fortified samples (Figure 3A). While, plotting the PC2 against the PC3, the distinction between M_10%_ and M_15%_ appears clearer (Figure 3B).

Descriptor groups (taste, odor, appearance and texture) are shown in Figure 3 using PC1 vs. PC2 (Figure 3C) and PC2 vs. PC3 (Figure 3D). The discrimination among the sample is highly influenced by the odor and taste, according to the PC1 and PC2, respectively. Overall, the odor is the main descriptor allowing the discrimination among all samples. However, the taste was an important discriminator between M_CT_ and M_15%_.

The correlation between the loading plots (Figure 3E,F) and the score plots (Figure 3A,B) highlights that the fortified muffins are closely connected with the descriptors linked to the texture, taste, odor and appearance (located in the left side of the plot); while the M_CT_ is closely related to taste and texture (Figure 3E,F), located in the right side of the plot. As reported by Liu et al. [35], all the assessors were asked to individually generate the sensory characteristics that best described the differences among the samples.

### 3.4. Bio-Preservation Effect

Inoculated (I) and non-inoculated (NI) muffin slices were stored and the fungal contamination of the muffins was observed throughout 21 days (Table 6). Overall, the fortification led to a lag of the fungal growth with a magnitude of differences according to the level of fortification. Indeed, after 7 days of storage, I-M_CT_ and I-M_5%_ showed *ca*. 20% of the surface colonized by *P. roqueforti* DPPMAF1, while at the same time I-M_10%_ presented *ca*. 10% of the surface contaminated. No contamination was found in I-M15%. Similar trend with higher level of contamination was found after 14 and 21 days of storage (Table 4). I-M_15%_ showed only *ca*. 20% of the surface colonized by *P. roqueforti* DPPMAF1 after 21 days of storage. When the spontaneous contamination (NI) was considered, the contaminated surface was lower than the corresponding inoculated muffins, regardless the level of inoculum, Moreover, NI-M_15%_ showed only ca. 10% of contaminated surface after 21 days of storage (Table 6). The fortification with 5% (wt/wt) of type-I sourdough did not produce any differences from the NI-M_CT_.

## 4. Discussion

Since the only treatment for CD is a lifelong GF diet [40], the production of GF foods has increased significantly in the last years and nowadays the GF market can boast a wide selection of products, such as bread, pasta, cookies and cakes. Nevertheless, defects in the final food product, due to the elimination of gluten, are still pressuring researchers and industries to produce food that could meet consumers’ demands in terms of sensory and nutritional quality, as well as sustainable costs. Nutritional deficiencies (e.g., dietary fiber, proteins and minerals) and excesses (e.g., fat and high GI) are the main concerns regarding the GF products. Indeed, enhancing the nutritional quality of GF products is an unquestionable and concomitant task along with the improvement of both their technological and sensory properties [41].

The use of GF ingredients, rich in nutritional components, flanked by the optimal technological process might represent a valuable option to produce GF products with optimal features and being accepted by the consumers. Maize and rice are the main cereals used as wheat-alternatives in bakery products, however the research and the industry are moving toward the rediscover of pseudo-, minor- and ancient- (amaranth, quinoa, teff, etc.) cereals due to their nutritional quality and interesting technological properties [10].

Teff contains many proteins (providing all essential amino acids, including lysine), slowly digestible complex carbohydrates (causing satiety), fibers (improving gut health) and more bioavailable minerals (among which calcium and iron) [7,42]. These properties make teff an interesting product for human consumption thus a functional food for the health development and prevention of diseases. Indeed, the interest in teff cultivation is spreading to many western countries of the world [43]. Nevertheless, due to the poorly appreciated sensory profile, biotechnological approaches, that is, sourdough fermentation, have been proposed to improve the aroma quality of teff baked goods [44,45].

In the present study, teff was used to produce type-I sourdough through back-slopping procedure in order to be used to fortify gluten-free teff muffins. According to the microbiological and biochemical characteristics, the type-I sourdough achieved the biochemical stability after 9 refreshments, with ratio between lactic acid bacteria and yeast stabilized at 100:1 in mature sourdough, as previously reported [46]. *L. plantarum* dominated since the beginning, however, mature sourdough was characterized by the concomitant presence of *Li. fermentum*. The prevalence of *Li. fermentum and L. plantarum* have already been reported as part of the dominant microbiota of teff flour [47] and sourdough [46]. Due to the lactic acid bacteria fermentation, increases of lactic and acetic acids concentrations were found, especially during the first three days of propagation. Nevertheless, if the former stabilized from t3, the latter varied through the propagation reaching the highest concentration after t9. The increase of the acetic acid might be ascribed to the appearance (between t9 and t12) of the strictly hetero-fermentative *Li. fermentum*. The balance between homo- and hetero-fermentative lactobacilli reflects on the organic acids released and in turn on the development of flavor and the microbial stability of the bread [48]. The FQ of the mature sourdough was 2.6 within the optimum range (2.0–2.7) as suggested by Hammes and Gänzle [49].

The mature sourdough was used to fortify GF muffins at three different level of inoculum (M_5%_, M_10%_ and M_15%_) and the characteristics were compared to those of a control made without sourdough (M_CT_). The biochemical characteristics of the sourdough reflected well on those of the final products, except for pH. The use of a chemical leavening agent to produce muffin might have buffered the acidity although concentrations of the organic acids > 20 mmol/kg were found (Table 1). The FQ, ranging from 2.3 to 2.9 suggested optimal sensory profile of the sourdough muffins [49].

Nevertheless, the quantification of the other flavor components as well as the identification of the sensory attributes which better describe the muffins have been performed. Overall, aldehydes and alcohols were found to be more concentrated in fermented samples than M_CT_. Several authors already reported that these compounds are generated by lipid oxidation in baked products [37,50], further highlighting that lipid oxidation is a process that begins during ingredients mixing and goes on until baking and during the storage [51]. Nevertheless, since the samples were subjected to the same conditions of kneading and baking, presumably the higher content of aldehydes and alcohols in sourdough containing-muffins could be due to the action of lactic acid bacteria and yeasts that during the fermentation process promote the generation of hexanal, 1-hexanol, hexanoic acid (as confirmed by the highest content in M_10%_ and M_15%_) [52]. Moreover, compounds having amino acids as precursor (e.g., 2-penthyl- and ehyl-octanoate and carbon sulfide) were more concentrated in sourdough containing-muffins due to the higher concentration of amino acids released during sourdough fermentation and the acidic environment [50]. Nevertheless, some of these compounds can also be formed during Maillard reaction, enhanced by free amino acids release [53,54]. A singular trend was observed for methyl ketones (C5-C9). As already reported in the literature [55], methyl ketones are formed from incomplete β-oxidation; the steps include β-oxidation of the released FFA to β-ketoacyl-CoA, which are then deacylated into β-ketoacids under the action of the thioesterases and then the keto acids are decarboxylated to methyl ketones. In fact, their formation can be related to β-oxidation of fatty acids carried out by both lactic acid bacteria and yeasts [55,56] as well as by seed enzymes [57]. They reached their maximum levels in M_5%_ muffins, pointing out the contribute of microbial β-oxidation to the volatile pattern of muffins, while decreasing where higher levels of sourdough were added (Table 4). This nonlinear trend could be attributed to an easier involvement of their carbonyl functional group in non-enzymatic browning, that is, Maillard reaction, occurring in higher extent in muffins with higher amounts of sourdough, as pointed also by color analysis showing a significant decrease of the luminosity index L* in the crust (Table S3).

Arendt et al. [58] indicated that most of the gluten-free bakery products on the market have very poor quality, particularly when compared to traditional wheat flour yeast bread, since they have reduced flavor and a crumbly and dry texture. Sourdough has been shown to improve overall bread quality, enhancing the textural properties and prolonging shelf life [59]. In this framework, the sensory evaluation and the textural parameters determination represent key points to develop a new gluten-free product. Our results from sensory evaluation highlighted that odor is the main descriptor to discriminate among M_CT_ and the fortified samples. Indeed, sourdough fermentation has widely been reported as suitable tool to improve the sensorial characteristics of GF baked goods [60] with lactic acid bacteria generating very specific aroma profiles and odorant compositions [59].

The highest values of firmness for the control and the samples M_5%_, agree with the those obtained from the image analysis, in fact firmness and porosity can be positively correlated, thus revealing information about the structure [61]. Porosity is caused by the production of the CO_2_ by yeast and some heterofermentative lactic acid bacteria and increases with fermentation [62].

The increase of the phenols extractability thanks to the acidic environment and the microbial enzymatic activity [63] led to higher radical scavenging activity in muffins containing the type-I sourdough. The effect was in accordance to the level of fortification (Table 2). Besides the nutritional value of the antioxidant compounds, from a technological point of view, such feature can contribute to the long-term oxidative stability of foods [64]. As regards the nutritional value of the muffins, high content of protein (>6%) and total dietary fibers (>3%) were found in all samples, thus suggesting the possible labels—“rich in protein” and “source of dietary fibers” [65].

The presence of high levels of insoluble fiber and high concentrations of antinutritional factors might be responsible for poor protein digestibility and amino acid availability [66]. Teff is rich in phytic acid, myo-inositol hexakisphosphate, which negatively affects the mineral and protein adsorption at the intestinal level [67]. This is, also, one of the major concerns in using teff to make staple foods [68]. As already suggested by the literature [69], the combined effect of the endogenous phytases activated through the acidification operated by lactic acid bacteria and the microbial activity led to significant decreases (up to 50% lower than control) of the content of the phytic acid in sourdough muffins (Table 3).

The proteolytic activity of the lactic acid bacteria led to increases of TFAA concentration, with relevant extents in the essential (2-times higher), hydrophobic (4-times) and aromatic (2-times) free amino acids (Figure 2). Hydrophobic and aromatic amino acids assist in radical scavenging and metal chelating activities. Amino acids with aromatic side groups are assumed to contribute to the strong radical scavenging activities of peptides [70]. The release of the FAA during sourdough fermentation also contributes to the enhancement of the nutritional value of sourdough and related food products due to their higher absorbance in the intestine [71,72]. Indeed, a 35% higher IVPD values were found in sourdough-containing muffins as compared to M_CT_.

The high content of fibers as well as the use of sourdough fermentation led to a decrease of the HI of sourdough-containing muffins as compared to the control. The synthesis of organic acids, especially lactic acid has been related to the decrease of the digestibility rate of the starch [73]. 

Food quality is a multivariate notion—taste, health and shelf-life need to be improved in parallel. Sourdough-containing muffins showed lower degree of fungal contamination when higher contents of sourdough were used for the fortification. Organic acids (i.e., acetic, phenyllactic) play an important role in terms of rope inhibition and prolonged shelf-life of baked products [74]. However, the release of antimicrobial compounds during sourdough fermentation (mainly peptides) have also to be considered [75].

## 5. Conclusions

A teff type-I sourdough, propagated through back-slopping procedure, has successfully been produced and used to prepare gluten-free muffins with high nutritional value, appreciable sensory profile and extended shelf-life. High content of TFAA (up to *circa* 1000 mg/kg) and proteins (>6%) and value of their IVPD (70%), as well as low HI (52%) and high concentration of fibers (> 3%) make the proposed muffins of great interest toward healthy and balance gluten-free diet. The role of the sourdough as bio-preserving agent for the extension of the product shelf-life perfectly meets the consumers ‘request for high-quality natural products.

## Figures and Tables

**Table 1:** Nutritional characterization of muffins: M_5%_, muffin containing 5% (wt/wt) type-I sourdough; M_10%_, muffin containing 10% (wt/wt) type-I sourdough; M_15%_, muffin containing 15% (wt/wt) of type-I sourdough; M_CT_, muffin made without type-I sourdough.

	M_5%_	M_10%_	M_15%_	M_CT_
*Proximate composition* *				
Moisture	21.18 ± 1.86 ^b^	25.43 ± 1.08 ^a^	26.92 ± 1.08 ^a^	21.33 ± 1.53 ^b^
Protein	7.67 ± 0.16 ^a^	7.78 ± 0.13 ^a^	7.62 ± 0.10 ^a^	7.75 ± 0.16 ^a^
Fat	22.52 ± 0.49 ^a^	22.67 ± 0.60 ^a^	23.03 ± 0.24 ^a^	22.96 ± 2.64 ^a^
*SFA*	2.14 ± 0.95 ^a^	2.76 ± 0.11 ^a^	2.79 ± 0.01 ^a^	2.75 ± 0.26 ^a^
*MUFA*	8.02 ± 0.18 ^a^	8.27 ± 0.21 ^a^	8.37 ± 0.11 ^a^	8.24 ± 0.94 ^a^
*PUFA*	11.81 ± 0.28 ^a^	11.65 ± 0.35 ^a^	11.88 ± 0.36 ^a^	11.91 ± 1.45 ^a^
Carbohydrates	43.46 ± 1.21 ^a^	37.49 ± 1.44 ^b^	36.28 ± 1.37 ^b^	42.42 ± 1.51 ^a^
Total dietary fibers	3.88 ± 0.77 ^a^	4.98 ± 0.89 ^a^	4.55 ± 1.22 ^a^	4.09 ± 1.09 ^a^
Ash	1.29 ± 0.26 ^b^	1.65 ± 0.17 ^a^	1.6 ± 0.09 ^a^	1.45 ± 0.50 ^ab^

Data are the means of three independent batches analysis ± standard deviations (*n* = 3). ^a,b^ Values in the same row with different superscript letters differ significantly (*p* < 0.05). * Data are expressed as g/100 g. SFA, saturated fatty acids; MUFA, Monounsaturated fatty acids; PUFA, polyunsaturated fatty acid. The ingredients and technological parameters used for daily sourdough back-slopping are reported in materials and methods.

**Table 2:** Biochemical characteristics and antioxidant activity of the teff muffins: M_5%_, muffin containing 5% (wt/wt) type-I sourdough; M_10%_, muffin containing 10% (wt/wt) type-I sourdough; M_15%_, muffin containing 15% (wt/wt) type-I sourdough; M_CT_, muffin made without type-I sourdough.

	M_5%_	M_10%_	M_15%_	M_CT_
pH	6.04 ± 0.05 ^b^	5.98 ± 0.04 ^b^	5.72 ± 0.05 ^c^	6.51 ± 0.05 ^a^
TTA (ml NaOH 0.1 M)	2.0 ± 0.4 ^c^	3.2 ± 0.6 ^b^	5.4 ± 0.5 ^a^	1.4 ± 0.3 ^c^
Lactic acid (mmol/kg)	25.9 ± 0.4 ^b^	30.4 ± 0.2 ^b^	48.4 ± 0.6 ^a^	n.d.
Acetic acid (mmol/kg)	9.8 ± 0.3 ^b^	10.5 ± 0.6 ^b^	20.8 ± 0.4 ^a^	n.d.
QF	2.6	2.9	2.3	n.d.
TFAA (mg/Kg)	824 ± 15 ^c^	987 ± 16 ^b^	1090 ± 18 ^a^	389 ± 14 ^d^
Peptide concentration (mg/100 g)	144 ± 20 ^a^	143 ± 15 ^a^	167 ± 15 ^a^	95 ± 15 ^b^
Total phenols (mmol/kg)	2.37 ± 0.05 ^b^	2.40 ± 0.04 ^b^	3.30 ± 0.05 ^a^	2.02 ± 0.03 ^c^
Radical scavenging (%) on ME	49.5 ± 0.5 ^b^	50.2 ± 0.3 ^b^	55.7 ± 0.4 ^a^	34.6 ± 0.5 ^c^
Radical scavenging (%) on WSE	44.9 ± 0.7 ^c^	46.6 ± 0.3 ^b^	50.6 ± 0.4 ^a^	40.6 ± 0.4 ^d^

TTA, total titratable acidity; QF, quotient of fermentation; TFAA, total free amino acids; ME, methanolic extract; WSE, water/salt extract. Data are the means of three independent batches analysis ± standard deviations (*n* = 3). ^a–d^ Values in the same row with different superscript letters differ significantly (*p* < 0.05). The ingredients and technological parameters used for daily sourdough back-slopping are reported in materials and methods.

**Table 3:** Phytic acid, IVPD and HI of the teff muffins: M_5%_, muffin containing 5% (wt/wt) type-I sourdough; M_10%_, muffin containing 10% (wt/wt) type-I sourdough; M_15%_, muffin containing 15% (wt/wt) type-I sourdough; M_CT_, muffin made without type-I sourdough.

	M_5%_	M_10%_	M_15%_	M_CT_
Phytic acid (mg/100 g)	116 ± 4 ^b^	116 ± 2 ^b^	95 ± 4 ^c^	223 ± 3 ^a^
IVPD (%)	70 ± 5 ^a^	75 ± 3 ^a^	78 ± 4 ^a^	50 ± 6 ^b^
HI (%)	62 ± 2 ^ab^	59 ± 1 ^b^	52 ± 3 ^c^	65 ± 3 ^a^
pGI	74 ± 2 ^a^	72 ± 1 ^a,b^	68 ± 3 ^b^	75 ± 3 ^a^

IVPD, in vitro protein digestibility; HI, starch hydrolysis index: pGI, predicted glycemic index. Data are the means of three independent batches analysis ± standard deviations (*n* = 3). ^a–c^ Values in the same row with different superscript letters differ significantly (*p* < 0.05). The ingredients and technological parameters used for daily sourdough back-slopping are reported in materials and methods.

**Table 4:** Volatile organic compounds (expressed as µg/g) of the teff muffins M_5%_, muffin containing 5% (wt/wt) type-I sourdough; M_10%_, muffin containing 10% (wt/wt) type-I sourdough; M_15%_, muffin containing 15% (wt/wt) type-I sourdough; M_CT_, muffin made without type-I sourdough.

Compounds	M_5%_	M_10%_	M_15%_	M_CT_
*Aldehydes*				
Hexanal	18.48 ± 2.67 ^b^	66.40 ± 4.20 ^a^	66.63 ± 5.21 ^a^	9.24 ± 3.37 ^c^
Octanal	5.23 ± 1.44 ^b^	14.29 ± 0.28 ^a^	15.67 ± 1.38 ^a^	14.45 ± 2.94 ^a^
2-Heptenal, (E)-	9.56 ± 1.67 ^b^	31.39 ± 6.45 ^a^	41.30 ± 15.32 ^a^	3.80 ± 0.47 ^c^
Nonanal	45.13 ± 3.18 ^b^	109.14 ± 13.70 ^a^	106.18 ± 13.62 ^a^	16.96 ± 3.33 ^c^
2-Octenal, (E)-	3.86 ± 1.31 ^b^	11.88 ± 3.32 ^a^	14.24 ± 4.25 ^a^	1.63 ± 0.42 ^c^
Benzaldehyde	2.57 ± 0.12 ^c^	5.33 ± 1.03 ^a^	7.06 ± 1.43 ^a^	3.00 ± 0.26 ^b^
2-Nonenal, (E)-	6.17 ± 0.57 ^b^	16.75 ± 5.33 ^a^	17.78 ± 3.33 ^a^	1.77 ± 0.31 ^c^
2,4-Decadienal, (E,E)-	n.d.	n.d.	7.35 ± 3.72 ^a^	n.d.
Phenylacetaldehyde	2.82 ± 0.30 ^b^	7.46 ± 0.83 ^a^	6.79 ± 1.11 ^a^	3.80 ± 1.87 ^b^
*Ketones and esters*				
2-Pentanone	83.99 ± 4.98 ^a^	21.14 ± 3.97 ^c^	21.89 ± 13.30 ^b,c^	34.33 ± 3.54 ^b^
2-Hexanone	36.96 ± 2.60 ^a^	10.80 ± 0.86 ^b^	11.27 ± 4.12 ^b,c^	13.98 ± 0.38 ^c^
2-Heptanone	232.58 ± 45.85 ^a^	66.73 ± 8.62 ^b^	62.87 ± 6.88 ^b^	46.39 ± 4.01 ^c^
3-Octanone	4.19 ± 0.43 ^b^	1.76 ± 0.07 ^c^	2.41 ± 0.80 ^c^	11.76 ± 4.08 ^a^
2-Octanone	101.68 ± 14.02 ^a^	19.53 ± 1.49 ^c^	15.15 ± 4.46 ^c^	28.64 ± 3.10 ^b^
6-methyl-5-hepten-2-one	n.d.	4.32 ± 1.22 ^a^	4.10 ± 1.04 ^a^	5.45 ± 1.61 ^a^
2-Nonanone	9.89 ± 0.72 ^a^	2.08 ± 0.18 ^b^	1.87 ± 0.35 ^b^	9.13 ± 1.10 ^a^
3-Octen-2-one, (E)-	n.d.	3.52 ± 1.21 ^a^	3.73 ± 0.81 ^a^	n.d.
Acetophenone	n.d.	3.87 ± 1.26 ^a^	5.17 ± 1.77 ^a^	n.d.
Ethyl octanoate	0.89 ± 0.20 ^c^	1.39 ± 0.21 ^b^	2.24 ± 0.55 ^a^	n.d.
Alcohols				
1-Hexanol	83.54 ± 4.71 ^c^	104.29 ± 7.19 ^b^	125.72 ± 10.65 ^a^	13.09 ± 2.50 ^d^
1-Octen-3-ol	37.07 ± 4.32 ^b^	20.02 ± 0.45 ^c^	21.29 ± 5.92 ^c^	94.17 ± 16.81 ^a^
1-Octanol	6.34 ± 1.85 ^b^	12.09 ± 0.84 ^a^	13.63 ± 1.38 ^a^	n.d.
Benzenemethanol	n.d.	3.36 ± 1.28 ^a^	2.76 ± 0.41 ^a^	n.d.
Phenylethyl alcohol	3.98 ± 0.33 ^b^	4.63 ± 0.43 ^b^	6.57 ± 0.63 ^a^	n.d.
*Acids*				
Propanoic acid	3.78 ± 0.28 ^a^	3.77 ± 0.19 ^a^	4.30 ± 0.33 ^a^	4.34 ± 0.89 ^a^
Hexanoic acid	4.62 ± 1.33 ^b^	17.74 ± 2.68 ^a^	20.59 ± 7.13 ^a^	2.84 ± 0.71 ^b^
Nonanoic acid	n.d.	n.d.	4.79 ± 1.59 ^a^	4.41 ± 0.85 ^a^
*Furans*				
Furan, 2-pentyl-	12.11 ± 0.66 ^b^	14.58 ± 0.25 ^a^	16.65 ± 2.10 ^a^	14.97 ± 1.13 ^ab^
2-Furanmethanol	n.d.	4.65 ± 0.71 ^a^	5.56 ± 0.74 ^a^	n.d.
2(3H)-Furanone, dihydro-5-pentyl-	9.34 ± 0.38 ^b^	7.77 ± 1.54 ^c^	12.01 ± 1.63 ^a^	3.18 ± 1.05 ^d^
*Sulfurs*				
Carbon sulfide	0.55 ± 0.28 ^b^	8.04 ± 1.37 ^a^	8.72 ± 1.38 ^a^	n.d.
*Others*				
dl-limonene	15.54 ± 0.80 ^b^	16.12 ± 1.37 ^b^	16.86 ± 1.95 ^b^	27.88 ± 6.72 ^a^
Phenol, 2,6-bis(1,1-dimethylethyl)-4-methyl	n.d.	n.d.	n.d.	4.48 ± 1.30 ^a^

Data are the means of three independent batches analysis ± standard deviations (*n* = 3). ^a–d^ Values in the same row with different superscript letters differ significantly (*p* < 0.05). The ingredients and technological parameters used for daily sourdough back-slopping are reported in materials and methods.

**Table 5:** Structural properties of the teff muffins: M_5%_, muffin containing 5% (wt/wt) type-I sourdough; M_10%_, muffin containing 10% (wt/wt) type-I sourdough; M_15%_, muffin containing 15% (wt/wt) type-I sourdough; M_CT_, muffin made without type-I sourdough.

	M_5%_	M_10%_	M_15%_	M_CT_
*Structural properties*				
Firmness (N)	29.6 ± 3.5 ^a^	19.5 ± 2.3 ^c^	22.4 ± 2.5 ^bc^	24.7 ± 1.1 ^b^
Springiness	0.89 ± 0.1 ^a^	0.89 ± 0.1 ^a^	0.88 ± 0.1 ^a^	0.88 ± 0.1 ^a^
Chewiness (N)	12.4 ± 1.4 ^a^	12.1 ± 3.6 ^a^	9.6 ± 1.2 ^a^	11.1 ± 0.4 ^a^
Cohesiveness	0.41 ± 0.01 ^b^	0.74 ± 0.01 ^a^	0.42 ± 0.01 ^b^	0.41 ± 0.01 ^b^
*Image analysis*				
Mean area (mm^2^)	0.72 ± 0.01 ^b^	0.94 ±0.08 ^a^	1.06 ±0.21 ^a^	0.66 ± 0.01 ^c^
Gas cells (n. cells/mm^2^)	220.4 ± 1.9 ^b^	161.4 ± 25.2 ^c^	141.3 ±44.2 ^c^	260.0 ± 21.2 ^a^

Data are the means of three independent batches analysis ± standard deviations (*n* = 3). ^a–c^ Values in the same row with different superscript letters differ significantly (*p* < 0.05). The ingredients and technological parameters used for daily sourdough back-slopping are reported in materials and methods.

**Table 6:** Fungal growth in teff muffins incubated at room temperature for 21 days. M_5%_, muffin containing 5% (wt/wt) type-I sourdough; M_10%_, muffin containing 10% (wt/wt) type-I sourdough; M_15_%, muffin containing 15% (wt/wt) type-I sourdough; M_CT_, muffin made without type-I sourdough. *Penicillium roquefortii* DPPMAF1 was used as indicator mold.

Day	Non-Inoculated				Inoculated			
	**M_5%_**	**M_10%_**	**M_15%_**	**M_CT_**	**M_5%_**	**M_10%_**	**M_15%_**	**M_CT_**
**7**	++	±	−	++	+	−	−	+
**14**	++++	++	±	++++	++	±	−	++
**21**	++++	+++	++	++++	+++	++	+	+++

Contamination was scored as follows: −: 0% of contamination of the surface; ±: 10% of contamination; +: 20% of contamination; ++: 40% of contamination; +++: 80% of contamination; ++++: 100% of contamination. The ingredients and technological parameters used for daily sourdough back-slopping are reported in materials and methods.

## Supplementary Materials

The following are available online at https://www.mdpi.com/2076-2607/8/8/1149/s1. Figure S1: Crumb image analysis of the teff muffins obtained by ImageJ software: MCT, muffin made without type-I sourdough (Panel A); M15%, muffin containing 15% (wt/wt) type-I sourdough (Panel B). Threshold (A′ and B′) and drawing (A″ and B″) are also reported; Table S1: Ingredients used for the manufacture of the teff muffins: M5%, muffin containing 5% (wt/wt) type-I sourdough; M10%, muffin containing 10% (wt/wt) type-I sourdough; M15%, muffin containing 15% (wt/wt) type-I sourdough; MCT, muffin made without type-I sourdough.; Table S2: List of the attributes used for the sensory analysis of the teff muffins; Table S3. Color parameters evaluation in crust and crumb of the teff muffins: M5%, muffin containing 5% (wt/wt) type-I sourdough; M10%, muffin containing 10% (wt/wt) type-I sourdough; M15%, muffin containing 15% (wt/wt) type-I sourdough; MCT, muffin made without type-I sourdough.
