# Supplementary material for: Teff Type-I Sourdough to Produce Gluten-Free Muffin
Source: Microorganisms. 2020 Jul 29;8(8):1149. doi: 10.3390/microorganisms8081149 (PMC7466135; doi:10.3390/microorganisms8081149)
Supplement: Supplementary file 1 [file microorganisms-08-01149-s001.pdf]

Table S1. Ingredients used for the manufacture of the teff muffins: $M_{5\%}$, muffin containing 5% (wt/wt) type-I sourdough; $M_{10\%}$, muffin containing 10% (wt/wt) type-I sourdough; $M_{15\%}$, muffin containing 15% (wt/wt) type-I sourdough; M_{CT}, muffin made without type-I sourdough.

Ingredient	$M_{5\%}$	$M_{10\%}$	$M_{15\%}$	M_{CT}
Teff flour	29.7 ± 0.4	27 ± 2	24.2 ± 0.2	32.44 ± 0.3
Sugar	17.8 ± 0.3	17.8 ± 0.3	17.8 ± 0.4	17.84 ± 0.3
Leavening agent*	1.1 ± 0.2	1.1 ± 0.3	1.1 ± 0.3	1.07 ± 0.2
Milk Powder	2.3 ± 0.4	2.3 ± 0.2	2.3 ± 0.2	2.27 ± 0.3
Tap Water	11.7 ± 0.2	9.4 ± 0.1	7.2 ± 0.2	13.95 ± 0.3
Oil	16.2 ± 0.3	16.2 ± 0.4	16.2 ± 0.3	16.2 ± 0.3
Eggs	16.2 ± 0.3	16.2 ± 0.3	16.2 ± 0.4	16.2 ± 0.4
Type-I Sourdough	5 ± 3	10 ± 2	15 ± 2	-

* Leavening agent, chemical leavening agent (PANEANGELI, Cameo s.p.a., Desenzano del Garda, Italy)

Table S2. List of the attributes used for the sensory analysis of the teff muffins.

Attributes			
Odor	***Taste***	***Texture***	***Appearance***
Eggs	Sweet	Firmness	Darkening
Burned	Sour	Crispness	Homogeneity
Damp	Fibres	Porosity	Porosity
Caramel	Yeast	Oily	Firmness
Vanilla	Bran	Chewiness	Graininess
Leafy	Oil	Graininess	Green (external)
Legumes	Vanilla	Springiness	Yellow (external)
Sponge cake	Tannic	Hardness	Green (internal)
Baking powder	Butter	Breaking strength	Yellow (internal)
Citrus	Damp	Gummy	Golden surface
Yogurt	Short pastry	Sandy	
Lactic	Sandy	Floury	
	Spicy		
	Salty		
	Smoked		
	Dry		
	Apple		

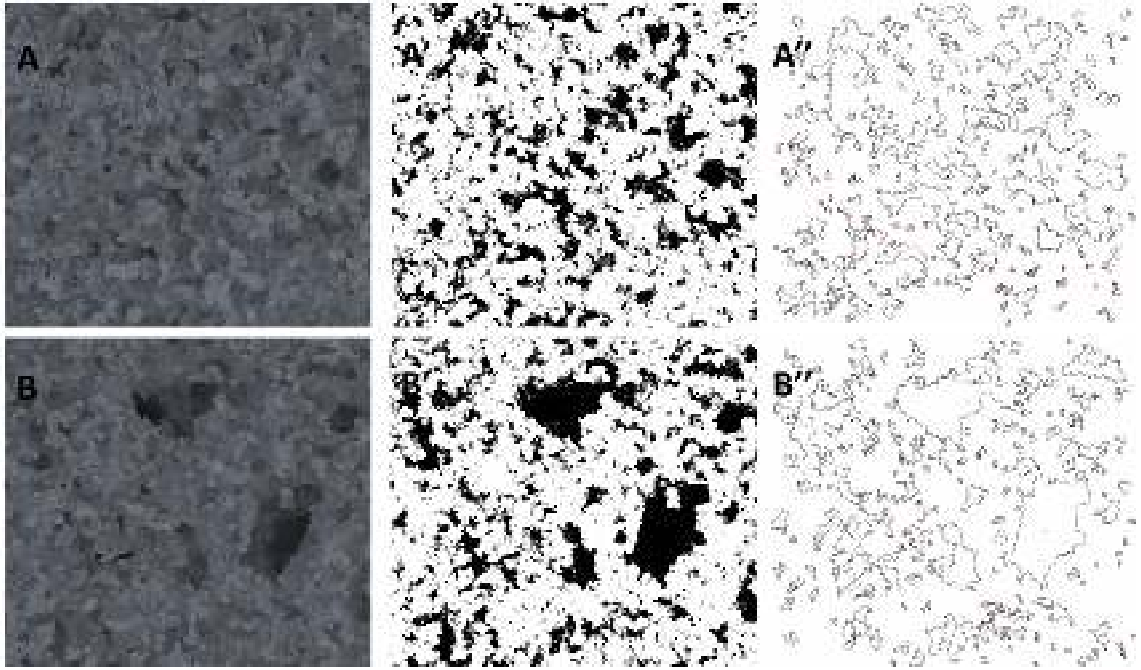

Figure S1. Crumb image analysis of the teff muffins obtained by ImageJ software: M_{CT}, muffin made without type-I sourdough (Panel A); $M_{15\%}$, muffin containing 15% (wt/wt) type-I sourdough (Panel B). Threshold (A′and B′) and drawing (A′′and B′′) are also reported.

Table S3. Color parameters evaluation in crust and crumb of the teff muffins :$M_{5\%}$, muffin containing 5% (wt/wt) type-I sourdough; $M_{10\%}$, muffin containing 10% (wt/wt) type-I sourdough; $M_{15\%}$, muffin containing 15% (wt/wt) type-I sourdough; M_{CT}, muffin made without type-I sourdough.

	**crust**			**crumb**		
Samples	*L**	*a**	*b**	*L**	*a**	*b**
$M_{5\%}$	46.9 ± 3.7^{ab}	10.1 ± 2.1^{a}	$29.5 \pm 4.1^{a,b}$	58.1 ± 1.5^{a}	4.5 ± 0.5^{a}	27.7 ± 0.3^{bc}
$M_{10\%}$	46.8 ± 2.9^{ab}	7.7 ± 1.5^{a}	$23.9 \pm 2.9^{a,b}$	59.7 ± 0.8^{a}	4.4 ± 0.2^{a}	29.1 ± 0.5^{ab}
$M_{15\%}$	40.8 ± 2.4^{b}	8.1 ± 0.8^{a}	24.2 ± 1.6^{b}	59.6 ± 1.2^{a}	4.5 ± 0.3^{a}	27.3 ± 0.4^{c}

M_{CT}	51.2 ± 3.9[a]	7.5 ± 1.7[a]	28.6 ± 1.6[a]	56.6 ± 2.3[a]	4.8 ± 0.5[a]	30.1 ± 0.7[a]

Data are the means of three independent batches analysis ± standard deviations (n = 3).[a-c] Values in the same column with different superscript letters differ significantly ($p<0.05$).
